# Immunological and Molecular Correlates of Disease Recurrence after Liver Resection for Hepatocellular Carcinoma

**DOI:** 10.1371/journal.pone.0032493

**Published:** 2012-03-02

**Authors:** Elisabetta Cariani, Massimo Pilli, Alessandro Zerbini, Cristina Rota, Andrea Olivani, Guido Pelosi, Claudia Schianchi, Paolo Soliani, Nicoletta Campanini, Enrico Maria Silini, Tommaso Trenti, Carlo Ferrari, Gabriele Missale

**Affiliations:** 1 Clinical Pathology-Toxicology, Ospedale S. Agostino-Estense, Modena, Italy; 2 Unit of Infectious Diseases and Hepatology, Azienda Ospedaliero-Universitaria of Parma, Parma, Italy; 3 Clinical Microbiology Laboratory, Department of Laboratory Medicine, Azienda Ospedaliera ASMN, Istituto di Ricovero e Cura a Carattere Scientifico, Reggio Emilia, Italy; 4 Department of General Surgery, Ospedale Santa Maria delle Croci, Ravenna, Italy; 5 Pathology Section, Department of Pathology and Laboratory Medicine, University of Parma, Parma, Italy; Singapore Institute for Clinical Sciences, Singapore

## Abstract

The definition of the risk of hepatocellular carcinoma (HCC) recurrence after resection represents a central issue to improve the clinical management of patients. In this study we examined the prognostic relevance of infiltrating immune cell subsets in the tumor (TIL) and in nontumorous (NT) liver (LIL), and the expression of immune-related and lineage-specific mRNAs in HCC and NT liver derived from 42 patients. The phenotype of infiltrating cells was analyzed by flow cytometry, and mRNA expression in liver tissue was examined by real-time reverse transcription (RT)-PCR. The tumor immune microenvironment was enriched in inhibitory and dysfunctional cell subsets. Enrichment in CD4+ T-cells and in particular CD4 and CD8+ memory subsets within TIL was predictive of better overall survival (OS) and time to recurrence (TTR). Increased programmed death ligand 1 (PDL1) mRNA content and higher prevalence of invariant NKT (iNKT) cells were associated with shorter OS and TTR, respectively. By combined evaluation of infiltrating cell subsets along with mRNA profiling of immune and tumor related genes, we identified the intratumoral frequency of memory T-cells and iNKT-cells as well as PDL1 expression as the best predictors of clinical outcome. HCC infiltrate is characterized by the expression of molecules with negative regulatory function that may favor tumor recurrence and poor survival.

## Introduction

Hepatocellular carcinoma (HCC) is a fatal disease occurring worldwide and developing mainly in patients with chronic liver disease [1]. Surgical resection is an important therapeutic option for small tumors, but the recurrence rate remains high and overall survival of resected patients at 5 years is dismal. The recurrence of HCC can be caused both by haematogenous dissemination of the primary tumor, mainly within the liver itself, and by the development of single or multiple de novo tumors arising in a chronically damaged liver.

The clinical and histopathological characteristics of HCC can only partially explain its prognostic heterogeneity. Several studies have postulated the existence of two main determinants for the risk of HCC recurrence: the molecular features of the tumor cells [2] and those of the liver environment [3], [4].

Considerable effort has been devoted to the identification of molecular profiles linked to HCC clinical features. Gene expression studies allowed the identification of HCC subclasses that share common molecular features and correlate with clinical parameters [5], [6]. Several lines of evidence support the relevance of hepatocyte differentiation [6], [7], [8], [9] and of immune response genes [5], [10], [11], [12] in the molecular classification of HCC, but the prognostic impact of the mRNA profile is still controversial.

The potential role played by tumor- and liver-infiltrating immune cells on the progression and recurrence of HCC is the subject of ongoing studies. Tumor-infiltrating lymphocytes are believed to inhibit tumor growth improving the prognosis of human malignancies, but the secretion of cytokines by infiltrating cells may also promote neovascularization and tumor dissemination [13], [14], [15].

The phenotypic characteristics of infiltrating immune cells appear a major determinant for the clinical outcome in several human tumors. Phenotyping of the intratumoral T-cells showed that high levels of memory T-cells correlated with better survival in colon cancer [16] and was associated with the expression of genes involved in Th1 adaptive immunity [17]. In earlier studies on HCC a T-cell infiltrate with predominance of CD8+ cells by immunohistochemistry was associated with improved 5-year survival rate [18]. More recently, the presence of lymphocyte infiltration together with a higher CD4∶CD8 T-cell ratio in HCC was reported as negative predictor of recurrence after liver transplantation [19]. In addition, a shift from a Th1 to a Th2-like profile has been postulated to occur in livers bearing metastatic HCC [3].

Despite evidences supporting a role of infiltrating cells in the surveillance of tumor progression, the poor prognosis of HCC reflects the failure of effective immune control of neoplastic growth. Cytotoxic T-cells (CTLs) infiltrating HCC show functional defects and incomplete activation associated with increased programmed death 1 (PD1) expression [20], [21] that is predictive of poorer disease outcome and postoperative recurrence [21]. Enriched programmed death ligand 1 (PDL1) expression was also found in tumors [21], [22] and linked to HCC prognosis [22]. Another mechanism implicated in failure of immune control against HCC progression is the suppression of effector cells by regulatory T-cells (Tregs) [23]. Increased circulating and/or intratumoral Tregs [22], [24], [25], [26], [27], as well as the balance between intratumoral CTLs and Tregs [28] have been associated with the outcome of HCC [29].

With the aim of a better comprehension of the characteristics of the intrahepatic lymphocyte infiltrate in patients with HCC, its possible role in disease outcome and its relation to histological and molecular characteristics of the tumor, we conducted a phenotypic analysis of tumor (TIL) and nontumorous (NT) liver (LIL) infiltrating lymphocytes, together with genomic profiling of immune related and lineage-specific mRNAs in HCC and NT liver.

## Results

### Characteristics of patients

The epidemiological, clinical and pathological characteristics of patients are depicted in Table 1. HCC nodules were 1 to 5 in the same patient and their size ranged from 30 to 50 mm in diameter. A poorly differentiated tumor tissue was present in the majority of patients. None of the patients showed macroscopic vascular invasion.

**Table 1:** Epidemiological, clinical and pathological characteristics of patients.

			Univariate				Multivariate		
Variable	Value		TTR		OS		TTR		OS
		HR		HR				HR	
		(95%CI)	p	(95% CI)	p		p	(95% CI)	p
**Age (≤68 vs >68 years)**	66.81±8.84	0.70	0.45	0.31	**0.03**	NA	NA	1.17	0.79
**mean ± SD**		(0.27–1.78)		(0.11–0.87)				(0.34–3.97)	
**Gender (M/F)**	31/11	0.61	0.36	0.09	**<0.001**	NA	NA	0.40	0.15
		(0.21–1.76)		(0.03–0.32)				(0.11–1.42)	
**HBsAg pos (n)**	4	1.31	0.7	0.5	0.32	NA	NA	NA	NA
		(0.34–5.06)		(0.13–1.97)					
**Anti-HCV pos (n)**	29	1.1	0.83	3.94	**<0.01**	NA	NA	3.82	0.25
		(0.46–2.63)		(1.45–10.73)				(0.38–38.44)	
**Alcohol use (n)**	11	0.87	0.74	0.29	**0.02**	NA	NA	0.41	0.45
		(0.37–2.05)		(0.1–0.8)				(0.04–4.19)	
**HCC (n = 1 vs >1)**	1 (1–5)	0.60	0.26	0.74	0.55	NA	NA	NA	NA
**median (range)**		(0.25–1.45)		(0.27–2.03)					
**Nodules size (≤50 vs >50 mm) mean ± SD**	57.62±25.6	1.07	0.87	1.80	0.23	NA	NA	0.55	0.29
		(0.47–2.42)		(0.70–4.6)				(0.18–1.66)	
**Tumour grade (1–2 vs 3)**		1.04	0.93	1.32	0.57	NA	NA	NA	NA
**median (range)**	3 (1–3)	(0.46–2.33)		(0.50–3.46)					
**Microvascular invasion pos (n)**	25	1.45	0.37	1.51	0.43	NA	NA	NA	NA
		(0.64–3.28)		(0.55–4.12)					
**TNM 1/2/3/4 (1 vs 2–4)**	10/21/10/1	0.59	0.24	1.32	0.59	1.03	0.93	NA	NA
		(0.25–1.42)		(0.46–3.79)		(0.39–2.71)			
**Lymphomononuclear infiltrate at histology (n)**	7	0.49	0.15	0.43	0.2	0.42	0.18	0.31	0.27
		(0.18–1.29)		(0.12–1.54)		(0.11–1.52)		(0.04–2.47)	

TTR: time to recurrence; OS: overall survival; HR: hazard ratio; CI: confidence intervals; NA: not applicable.

The median overall survival (OS) was 84 months, while the median time to recurrence (TTR) was 24 months (Fig. S1). Five patients, deceased within 3 months from surgery, were only included in the overall survival analysis but not in the prognostic analysis of TTR and OS regarding all immunological and molecular parameters.

Kaplan-Meier survival analysis of all epidemiological, clinical and pathological characteristics listed in Table 1 showed significantly longer OS for patients younger than 68 years and for alcohol abusers. Shorter OS was associated with both hepatitis C virus (HCV) infection, and female sex. However women of our cohort were significantly older (p<0.005), explaining this unexpected result. Multivariate analysis failed to identify any independent parameter associated with TTR or OS (Table 1).

### Differential distribution and clinical correlates of cell subsets and mRNAs in HCC and nontumorous liver tissue

**Figure 1:**
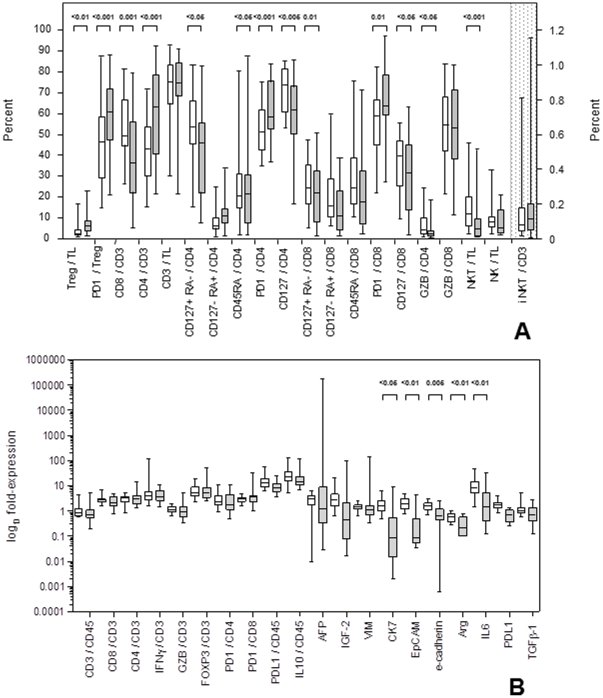
Differential distribution of infiltrating cell subsets and mRNAs between nontumorous and tumorous liver tissue samples. A: infiltrating cell subsets B: mRNAs; white bars: nontumorous and grey bars: tumorous liver tissue samples. Significance levels are shown on top of each panel. The right y axis of the upper panel refers to iNKT/CD3.

The distribution of cell subsets and of some of the analyzed mRNAs was significantly different among HCC and nontumorous liver tissues. HCC infiltrating cells were enriched in Tregs (CD4+, CD25+, FoxP3+), PD1+/CD4+ or PD1+/CD8+ T-cells and PD1+ Tregs. The frequency of the whole CD8+ T-cell population within the total lymphocyte infiltrate was increased in nontumorous liver tissue while CD4+ T-cells were more represented in HCC tissue. Memory (CD127+/RA−) CD4+ and CD8+ T-cell subsets, granzyme B (GZB)+/CD4+ T-cells and NKT-cells were all enriched in the NT liver tissue (Fig. 1A). Although CD8/CD3 mRNA was more abundant in NT liver and PD1/CD8 mRNA in HCC, the differences did not reach statistical significance. mRNAs encoding cytokeratin 7 (CK7), EpCAM, e-cadherin, arginase and interleukin 6 (IL6) were all significantly more represented in NT liver tissue than in HCC (Fig. 1B).

Among the histological characteristics of tumors, microvascular invasion was accompanied by higher frequency of Tregs (CD25+ FoxP3+/CD3+, p<0.05) and lower levels of e-cadherin (p<0.05) mRNA expression in HCCs; higher tumor grade ( = 3) was associated with higher expression of CD45 (p<0.05) and higher frequency of Tregs (CD25+ FoxP3+/CD3+, p<0.05) in HCC when compared to lower grade; presence of histological lymphomononuclear infiltrate was detected in HCCs with higher alpha fetoprotein (AFP) expression (p<0.05). The levels of transforming growth factor ß1 (TGFß1) mRNA in tumorous tissue correlated with expression of IL6 (p<0.001), vimentin (VIM) (p = 0.01) and PDL1 (p<0.05) (data not shown).

### Tumor infiltrating lymphocyte subsets and mRNA expression levels in the prediction of post-resection outcome

Levels of mRNA expression and frequencies of lymphocyte subsets in TIL and LIL were evaluated by Kaplan-Meier survival curves for their association with TTR and OS. For each variable, median levels were used as a cut-off value to analyze the association with survival.

**Figure 2:**
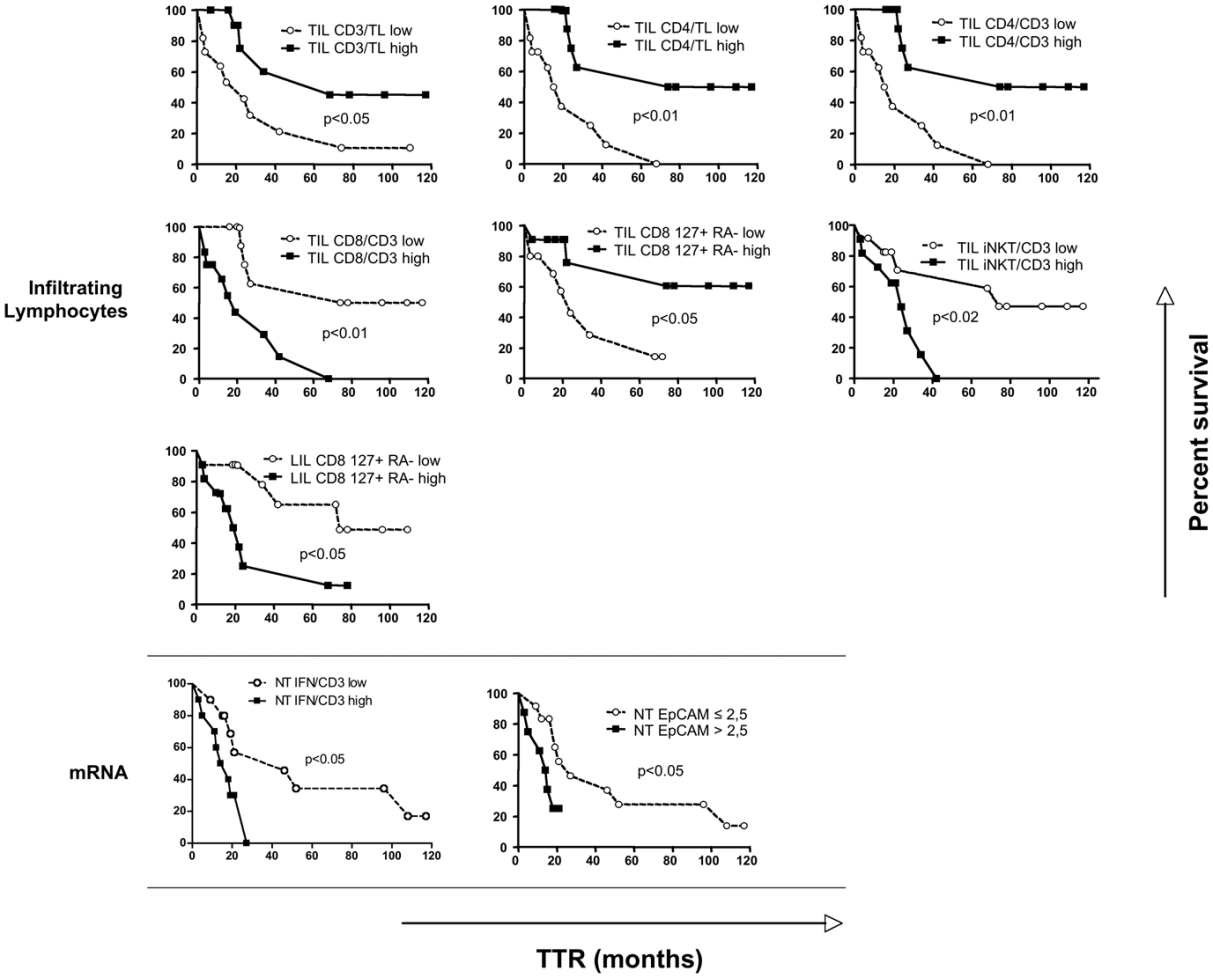
Kaplan-Meier curves of time to recurrence (TTR) among HCC patients. Prognostic significance of the frequency of tumor (TIL) or nontumorous liver infiltrating lymphocytes (LIL) are shown in the upper part of the figure. mRNA expression in nontumourus liver of IFN-γ and EpCAM is shown in the lower part of the figure. A specific cut-off (above or below 2.5-fold reference normal liver) was applied to EpCAM mRNA. TL: total lymphocytes; low, high: lower or higher than median value. P values were determined by the log-rank test.

**Figure 3:**
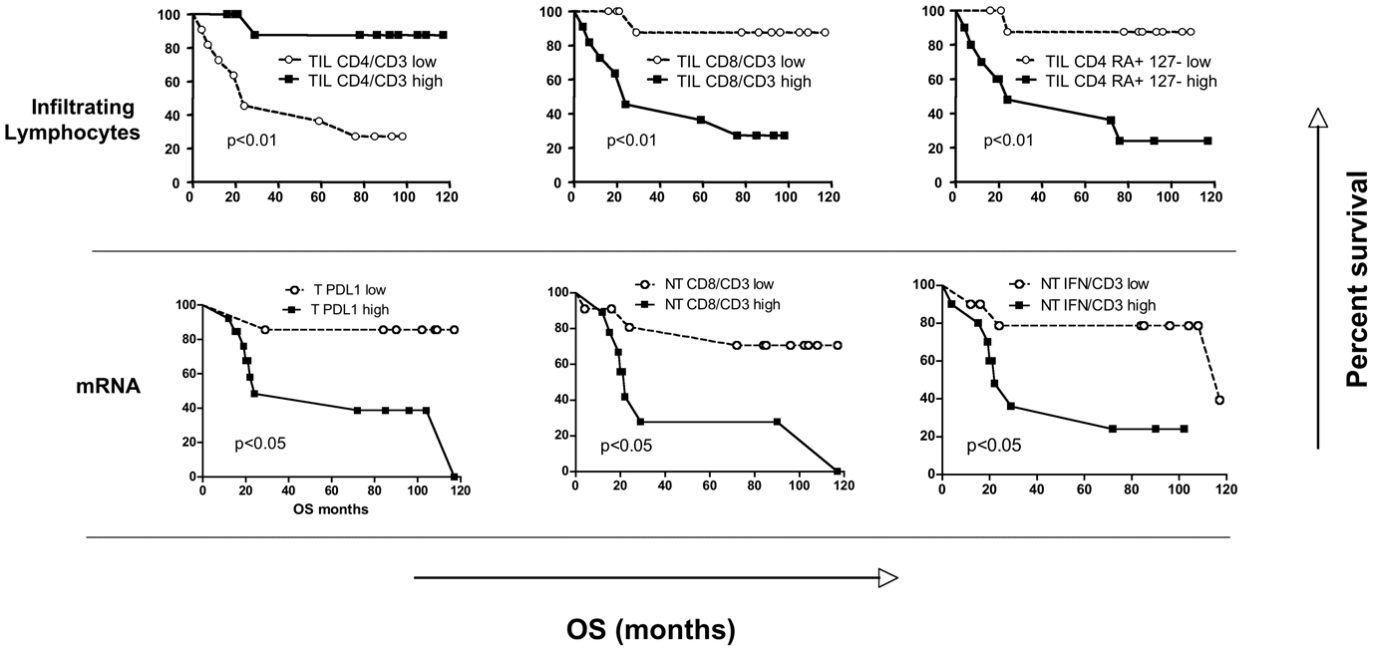
Kaplan-Meier curves of overall survival (OS) among HCC patients. Prognostic significance of the frequency of tumor infiltrating lymphocytes (TIL) are shown in the upper part of the figure. mRNA expression of PDL1 in tumor and of CD8 and IFN-γ in nontumorous liver is shown in the lower part of the figure. Low, high: lower or higher than median value. P values were determined by the log-rank test.

In the tumorous tissue, the frequency of CD3 and CD4+ cells within the whole lymphocyte population were significantly associated with longer TTR (Fig. 2). Furthermore CD4+/CD3+ cells were more represented in patients with better TTR and OS (Figs. 2 and 3, respectively). An opposite association was shown for CD8+/CD3+ cell subset, which was predictive of shorter TTR and OS. Further phenotypic characterization, by staining for CD127 and CD45RA, however showed that higher levels of differentiation to a memory phenotype were associated with longer TTR for CD8 (CD8+ CD127+RA− cells). By contrast, a higher frequency of effector CD4+ cells (CD4 RA+127−) was associated with shorter OS (Fig. 3).

Limiting the survival analysis to the anti-HCV positive patients, the same trends were confirmed as in the whole population, maintaining significant association with longer TTR for higher frequency of CD8 CD127+RA− cells in TIL (p<0.05; not shown).

Among the remaining lymphocyte subsets represented in TIL, only iNKT cells were significantly predictive of shorter TTR (Fig. 2). Because of the limited amount of stored TIL, further characterization of this lymphocyte subset could be performed in only 5 patients, by staining iNKT-cells for CD4 and CD8 markers. Most iNKT/CD3 cells were CD4+ (86.3±14.3%), suggesting that regulatory or Th2 iNKT-cells may be enriched in TIL of patients with worse outcome. A prevalent CD4+ iNKT-cell phenotype was also present in the peripheral blood compartment (67.7±25.1) of the same patients but frequencies of iNKT/CD3 and CD4+ iNKT-cells in the peripheral compartment were not significantly associated with outcome (either TTR or OS) (data not shown). In contrast to what observed within the tumor, patients with a higher frequency of memory CD8+ T-cells in the nontumorous tissue had a shorter TTR (Fig. 2).

**Figure 4:**
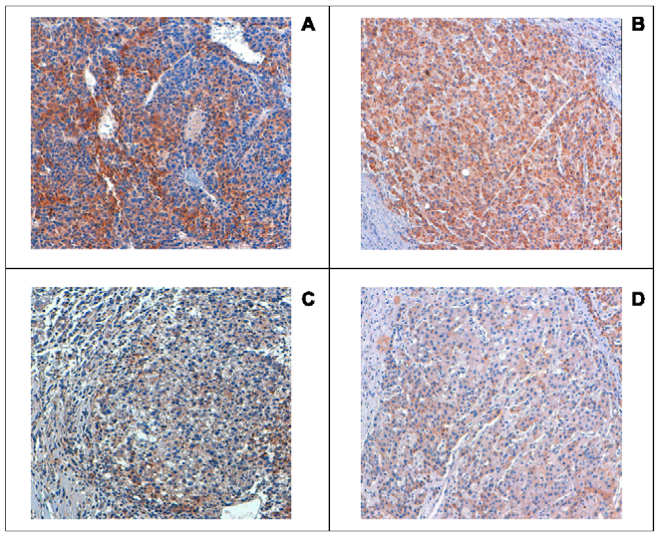
PDL1 immunohistochemical staining. Four representative cases with high (A and B) and low (C and D) protein expression are shown. PDL1 is mainly expressed by hepatoma cells rather than infiltrating lymphomononuclear cells and stromal cell elements.

Shorter TTR was observed when increased interferon γ (IFN γ) and EpCAM (>2.5-fold) mRNAs were detected in NT (Fig. 2). Higher PDL1 mRNA content in HCC, as well as CD8 and IFNγ mRNAs in NT liver higher than median levels were associated with shorter OS (Fig. 3). The analysis of tissue sections by immunohistochemical staining revealed that PDL-1 expression was mainly expressed in tumor cells rather than in nonparenchymal cells (Fig. 4).

## Discussion

Several lines of evidence suggest that the characteristics of both tumor cells and infiltrating immune cells may represent major determinants for the clinical outcome of HCC. Large-scale gene expression studies have converged in the identification of three major molecular profiles, characterized by high proliferation and chromosomal instability, by activation of the Wnt signaling pathway, and by IFN signalling due to tumor-infiltrating cells, respectively [5], [6], [7], [8], [9], [10], [30], [31].

In most of previous reports, tumor and infiltrating cells have been investigated independently and by different approaches: infiltrating cell phenotype was evaluated in situ by immunohistochemistry or on separated infiltrating cells by flow cytometry [18], [19], [20], [21], [22], [23], [24], [25], [26], [27], [28], whereas mRNA expression was analyzed by microarray or quantitative RT-PCR [5], [6], [7], [8], [9], [10], [31]. In this study we used an integrated approach including both phenotypic analysis of infiltrating cells by flow cytometry and mRNA quantitation by RT-PCR.

Our study shows, in agreement with previous reports [12], [20], [21], [26], an important difference in the immune microenvironment of HCC compared to nontumorous liver, since inhibitory and dysfunctional cell subsets such as Tregs and PD1+ cells were prevalent in the tumor. However the differential distribution of CD8+, CD4+, FOXP3+, and PD1+ cell subsets between HCC and nontumorous liver was not confirmed by mRNA quantitation. Individual discrepancies between mRNA and protein levels might be explained by cellular control mechanisms operating at transcriptional and/or at translational level. In addition, the overall mRNA amount is quantified regardless of the expressing cell type(s), whereas phenotypic characterization detects the abundance of a cell subset within the total infiltrating cell population.

Previous studies showed that a better outcome of HCC can be associated with the presence of lymphomononuclear infiltrate, low circulating and/or intratumoral Tregs, and a positive balance between intratumoral CTLs and Tregs [22], [24], [25], [26], [27], [28], [29]. The lack of a significant association between Tregs frequency and clinical outcome in our series may rely on the fact that more advanced HCCs were included in previous studies (TNM stage III and IV, Chinese anticancer association stage III, Japanese general rules for primary liver cancer stage III and IV) [24], [25], [26], [27], [28], that included cases with macrovascular tumor thrombosis and distant metastasis that were not present in our patients, with the exception of a single case presenting direct infiltration of the transverse colon wall. Moreover frequency of regulatory T cells within the tumor was significantly lower in TNM 1 patients of our cohort (p<0.01, data not shown). Indeed, an association between Tregs prevalence and HCC stage has been reported [25], [26], [28]. In our series, tumors with microvascular invasion were enriched in Tregs, in agreement with previous reports [19], [28] and had lower expression of mRNA encoding e-cadherin, an adhesion molecule frequently inactivated during the cancer invasion process [32].

In our cohort a better representation of CD4 and CD8+ memory subsets was present in tumors with a more benign outcome and was predictive of better overall survival and time to recurrence, indirectly suggesting that effector CD4 and CD8+ cells, that were enriched in tumors with poor outcome, were likely represented by dysfunctional T-cells.

Tumors with increased levels of AFP mRNA and high histological grade were characterized by more abundant inflammatory infiltrate and the infiltrate of high-grade tumors was enriched in Tregs. Consistent with these observations is the probability that less differentiated (i.e. higher grade) HCCs elicit the host immune response, possibly directed against differentiation-related antigens, such as AFP, but that effectiveness of the anti-tumor response is impaired by the high prevalence of Tregs within infiltrating cells. The relative abundance of PD1+ cells observed within the tumor infiltrate might contribute to the inhibition of tumor-specific T-cells. In this context the correlation between the expression of PDL1 and TGFß1 is suggestive of synergic mechanisms of impaired tumor-specific T-cell response in the same tumors.

Interestingly, in our study iNKT-cells were enriched in HCCs with poor clinical outcome. CD4+ iNKT-cells are known to provide a regulatory function by Th2 cytokine production and are implicated in inhibiting expansion of tumor antigen-specific CD8+ T-cells. The CD4+ iNKT-cells have been found to be enriched in intrahepatic malignant tumors [33] and could represent a new target for immunotherapy. Thus, the prevalent CD4+ phenotype of iNKT detected in our patients may contribute, along with CD4+CD25+FoxP3+ cells, to create a negative intratumoral microenvironment responsible for a dysfunctional CD8 cell response.

A limited number of studies found a relationship between gene expression profile of the tumor and survival outcome, reporting poor prognosis in tumors with gene signatures related to cell proliferation and to fetal-progenitor hepatic lineages [7], [8], [30]. In our analysis, only PDL1 among mRNAs expressed in HCCs was associated significantly with poor outcome, in agreement with previous observations of a tumor microenvironment favouring T-cell dysfunction and apoptosis [20], [21], [22]. The gene expression profile of nontumorous liver tissue has also been implicated in the prognosis of HCC [3], [4], [31]. We identified IFNγ mRNA in NT tissue as predictor of both shorter TTR and OS, while CD8/CD3 was only related to shorter OS. These results suggest that an inflammatory liver microenvironment may favor tumor recurrence, in agreement with recent results [4].

In conclusion, by a combined qualitative evaluation of infiltrating T and NK cell subsets along with mRNA profiling of immune and tumor related genes, we showed that intratumoral enrichment of memory CD4 and CD8 T-cells may be predictive of a better clinical outcome, while iNKT-cell frequency and PDL1 expression were associated with shorter time to recurrence and overall survival, respectively. Comparison of lymphocyte subset frequencies between liver and tumor provide evidence that HCC are characterized by the expression of molecules with negative regulatory function that may favor tumor recurrence and poor survival.

## Materials and Methods

### Patients and samples

We evaluated 42 HCC patients that underwent liver surgery at the University Hospital of Parma, Italy in the period 2001–2003. These patients were diagnosed with HCC by ultrasonography, computed tomography and magnetic resonance imaging in selected cases. Four patients had been previously treated by surgery and radiofrequency ablation, and underwent surgery because of HCC recurrence. Liver function was assessed by the Child-Pugh grading and all patients were within Child A scoring. Serological markers of hepatitis B virus (HBV) and HCV were performed for all patients as well as anti-human immunodeficiency virus that was negative for all cases. All patients were treated and received the same postoperative care by the same team of surgeons, and had postoperative follow-up every three months for the first 2 years, and every 6 months thereafter. The clinicopathological features of the patients analyzed were age, gender, number of tumor nodules, cumulative tumor size (sum of the main nodules diameters), etiology of the liver disease, microvascular venous infiltration, presence of lymphomononuclear infiltrate at histology, cellular differentiation according to the Edmondson classification (Table 1). The study was approved by the local ethical committee (Comitato Etico Indipendente (IRB/IEC) dell'Azienda Ospedaliera di Parma, Parma, Italy), and patients gave written informed consent to participate in the study.

### Biological samples

Liver and tumor specimens were collected in the operating room. Nontumorous liver tissue was always derived from the same liver segment of the HCC nodule with a free margin from the tumor tissue. Tumor and liver infiltrating lymphocytes were freshly derived, vitally frozen and stored in liquid nitrogen for later analysis. For subsequent mRNA extraction and amplification specimens were snap-frozen directly in the operating room. Unfortunately, this was not possible for all specimens because of logistics and the diagnostic needs of pathology; for this reason paired analysis of infiltrating lymphocytes and mRNA expression was available only for 13 subjects.

### Isolation of liver and tumor infiltrating lymphomononuclear cells (LIL, TIL)

Tissue samples of HCC and nontumorous liver were obtained at surgery and immediately stored in RPMI solution. For the extraction of lymphomononuclear infiltrate, contaminating blood was promptly removed by repeated washing with fresh RPMI using a syringe and a 23 gauge needle. Tissues were then dissected into small portions with a surgical blade, underwent a new step of RPMI washing and finally were digested with collagenase (1 mg/ml) and DNAse (25 mg/ml) (Sigma, St. Louis, MO, USA) for 1 h at 37°C. Thereafter, tissue suspension was filtered with a cell strainer (70 µm) (Becton Dickinson, CA, USA) and resuspended in complete medium (RPMI-1640 with 25 mM Hepes, 2 mM L-glutamine, 50 mg/ml gentamycin and 10% heat-inactivated human serum). This approach yielded 1–5×10^6^ infiltrating lymphomononuclear cells.

### Phenotypic analysis of T and NK-cells in liver and tumor compartments

For phenotypic characterization of lymphocyte populations, cells were stained with monoclonal antibodies (MoAb) anti-PD1, -forkhead/winged helix scurfy (FoxP3), -CD4, -GZB, -CD25, -CD8, -CD127, -CD45RA, -CD3, -Vα 24, -Vß11, -CD56. Intracellular staining for FoxP3 and GZB was performed using Fixation/Permeabilization reagents (e-Bioscience, San Diego, CA) according to manufacturer's protocol.

To assess the phenotypic and functional characteristics of lymphocyte populations derived from the different districts, the following panels of MoAb were used:

Panel 1: anti-PD1/-FoxP3/-CD4/-GZB/-CD25/-CD8 for Tregs identification, GZB content and PD1 expression on T and Tregs.
Panel 2: anti-PD1/-CD4/-CD127/-CD45RA/-CD3/-CD8 for characterization of memory and effector T-lymphocytes.
Panel 3: anti-Vα 24/-Vß11/-CD3/-CD56/-CD8/-CD4 for NK and NKT subsets.

### RNA extraction, cDNA synthesis and quantitative analysis of selected mRNAs by RT-PCR

Tissue specimens for subsequent RNA extraction were snap-frozen in liquid nitrogen just after resection. Total RNA was isolated from tumors and surrounding tissue by Trizol reagent (Invitrogen, Carlsbad, CA) or RNAeasy Mini-kit (Qiagen, Hilden, Germany). Random-primed cDNA first strand was synthesized by Superscript VILO (Invitrogen) and amplified in duplicate by TaqMan Gene-Expression Assays and Taqman Gene-Expression Master Mix (Applied Biosystems, Foster City, CA) on a Rotor Gene 3000 (Corbett Research, Sydney, Australia). Each sample was amplified in duplex with the internal reference human hypoxanthine-guanine phosphorybosyl transferase (HPRT1) using Human HPRT1 MGB Taqman assay (Applied Biosystems). Amplification efficiency of individual reactions, calculated by the Rotor Gene software, was automatically used as a correction factor for results and allowed the exclusion from analysis of reactions showing low efficiency.

A relative quantitation approach was applied by comparing the level of target gene expression between a sample and a reference material, i.e. normal donor peripheral blood mononuclear cells for immune response-related genes, or normal liver RNA (Ambion Corp., The Woodlands, TX) for lineage-related genes. The endogenous control HPRT1 was used to normalize input RNA amounts. Targets for the quantitation of gene expression were selected among molecular biomarkers potentially related to HCC prognosis [3]–[12] and included differentiation-related mRNAs expressed by fetal/progenitor liver cells, as insulin-like growth factor 2 (IGF2), AFP, EpCAM, CK7, VIM; genes potentially involved in HCC growth and invasiveness as e-cadherin, an adhesion molecule, and arginase. In addition, genes involved in adaptive and innate immune response as CD8, GZB, IFNγ, IL6, FoxP3, PD1, PDL1 and TGFß1 were also examined.

The results (target levels normalized to endogenous control levels) were expressed as a fold-change or a fold-difference compared to the reference tissue. mRNA level of immune response genes was further normalized to mRNA encoding CD3, CD4 or CD8 as appropriate. Since PDL1 is expressed both by infiltrating and in nonimmune cells, its mRNA was included in the analysis with or without prior normalization to CD45, considered as representative of the total liver infiltrate.

### Immunohistochemistry

Immunohistochemical staining of formalin fixed, paraffin-embedded tissue sections was performed on 15 HCC samples. The rabbit polyclonal Anti-CD274 (Abcam, Cambridge, MA), 5 µg/ml, was used as primary antibody. Heat-mediated antigen retrieval was performed with sodium citrate buffer (pH6) for 20 minutes; incubation was at room temperature for 15 minutes. Slides were developed with a horseradish peroxidase-conjugated compact polymer system (Advance HRP Kit, Dako) using diaminobenzidine as chromogen and counterstained with haematoxylin. Negative controls consisted of substituting normal serum for the primary antibody.

### Statistical analysis

Statistical analysis was performed by Graph-Pad Prism 5.04 Software. The differences between groups of continuous variables were analyzed by Mann-Whitney test or Wilcoxon matched pairs test, as appropriate, whereas categorical variables were compared by Fisher's exact test. Correlation was analyzed by the Spearman correlation coefficient. Survival curves were estimated by the Kaplan-Meier method and compared by log-rank test. Cox regression technique was performed for multivariate analysis. For the selection of variables to be included in the multivariate model we chose variables presenting p<0.25 at the univariate analysis. A p value<0.05 (two-tailed) was considered significant.

## Supporting Information

Figure S1 **Percent survival of the whole study population after surgery.** OS: overall survival; TTR: time to recurrence.
(TIF)

## References

[1] El-Serag HB, Rudolph KL (2007). Hepatocellular carcinoma: epidemiology and molecular carcinogenesis.. Gastroenterology.

[2] Thorgeirsson SS, Lee JS, Grisham JW (2006). Molecular prognostication of liver cancer: end of the beginning.. J Hepatol.

[3] Budhu A, Forgues M, Ye Q-H, Jia HL, He P (2006). Prediction of venous metastases, recurrence, and prognosis in hepatocellular carcinoma based on a unique immune response signature of the liver microenvironment.. Cancer Cell.

[4] Hoshida Y, Villanueva A, Kobayashi M, Peix J, Chiang DY (2008). Gene expression in fixed tissues and outcome in hepatocellular carcinoma.. N Engl J Med.

[5] Chiang DY, Villanueva A, Hoshida Y, Peix J, Newell P (2008). Focal gains of VEGFA and molecular classification of hepatocellular carcinoma.. Cancer Res.

[6] Hoshida Y, Nijman SM, Kobayashi M, Chan JA, Brunet JP (2009). Integrative transcriptome analysis reveals common molecular subclasses of human hepatocellular carcinoma.. Cancer Res.

[7] Lee JS, Chu IS, Heo J, Calvisi DF, Sun Z (2004). Classification and prediction of survival in hepatocellular carcinoma by gene expression profiling.. Hepatology.

[8] Lee JS, Heo J, Libbrecht L, Chu IS, Kaposi-Novak P (2006). A novel prognostic subtype of human hepatocellular carcinoma derived from hepatic progenitor cells.. Nat Med.

[9] Boyault S, Rickman DS, de Reyniès A, Balabaud C, Rebouissou S (2007). Transcriptome classification of HCC is related to gene alterations and to new therapeutic targets.. Hepatology.

[10] Breuhahn K, Vreden S, Haddad R, Beckebaum S, Stippel D (2004). Molecular profiling of human hepatocellular carcinoma defines mutually exclusive interferon regulation and insulin-like growth factor II overexpression.. Cancer Res.

[11] Sakai Y, Honda M, Fujinaga H, Tatsumi I, Mizukoshi E (2008). Common transcriptional signature of tumor-infiltrating mononuclear inflammatory cells and peripheral blood mononuclear cells in hepatocellular carcinoma patients.. Cancer Res.

[12] Chew V, Tow C, Teo M, Wong HL, Chan J (2010). Inflammatory tumor microenvironment is associated with superior survival in hepatocellular carcinoma patients.. J Hepatol.

[13] Disis ML (2010). Immune regulation of cancer.. J Clin Oncol.

[14] Budhu A, Wang XW (2006). The role of cytokines in hepatocellular carcinoma.. J Leukoc Biol.

[15] Li J, Lau GK, Chen L, Dong SS, Lan HY (2011). Interleukin 17A promotes hepatocellular carcinoma metastasis via NF-kB induced matrix metalloproteinases 2 and 9 expression.. PLoS One.

[16] Pagès F, Berger A, Camus M, Sanchez-Cabo F, Costes A (2005). Effector memory T-cells, early metastasis, and survival in colorectal cancer.. N Engl J Med.

[17] Galon J, Costes A, Sanchez-Cabo F, Kirilovsky A, Mlecnik B (2006). Type, density, and location of immune cells within human colorectal tumors predict clinical outcome.. Science.

[18] Wada Y, Nakashima O, Kutami R, Yamamoto O, Kojiro M (1998). Clinicopathological study on hepatocellular carcinoma with lymphocytic infiltration.. Hepatology.

[19] Unitt E, Marshall A, Gelson W, Rushbrook SM, Davies S (2006). Tumor lymphocytic infiltrate and recurrence of hepatocellular carcinoma following liver transplantation.. J Hepatol.

[20] Gehring AJ, Ho ZZ, Tan AT, Aung MO, Lee KH (2009). Profile of tumor antigen-specific CD8 T-cells in patients with hepatitis B virus-related hepatocellular carcinoma.. Gastroenterology.

[21] Shi F, Shi M, Zeng Z, Qi R-Z, Liu Z-W (2011). PD-1 and PD-L1 upregulation promotes CD8(+) T-cell apoptosis and postoperative recurrence in hepatocellular carcinoma patients.. Int J Cancer.

[22] Gao Q, Wang XY, Qiu SJ, Yamato I, Sho M (2009). Overexpression of PDL1 significantly associates with tumor aggressiveness and postoperative recurrence in human hepatocellular carcinoma.. Clin Cancer Res.

[23] Unitt E, Rushbrook SM, Marshall A, Davies S, Gibbs P (2005). Compromised lymphocytes infiltrate hepatocellular carcinoma: the role of regulatory T-cells.. Hepatology.

[24] Yang XH, Yamagiwa S, Ichida T, Matsuda Y, Sugahara S (2006). Increase of CD4+ CD25+ regulatory T-cells in the liver of patients with hepatocellular carcinoma.. J Hepatol.

[25] Kobayashi N, Hiraoka N, Yamagami W, Ojima H, Kanai Y (2007). FOXP3+ regulatory T-cells affect the development and progression of hepatocarcinogenesis.. Clin Cancer Res.

[26] Fu J, Xu D, Liu Z, Shi M, Zhao P (2007). Increased regulatory T-cells correlate with CD8 T-cell impairment and poor survival in hepatocellular carcinoma patients.. Gastroenterology.

[27] Zhou J, Tong D, Pan W, Zhu L-Y, Li L (2009). Increased intratumoral regulatory T-cells are related to intratumoral macrophages and poor prognosis in hepatocellular carcinoma patients.. Int J Cancer.

[28] Gao Q, Qiu SJ, Fan J, Zhou J, Wang XY (2007). Intratumoral balance of regulatory and cytotoxic T-cells is associated with prognosis of hepatocellular carcinoma after resection.. J Clin Oncol.

[29] Wilke CM, Wu K, Zhao E, Wang G, Zou W (2010). Prognostic significance of regulatory T cells in tumor.. Int J Cancer.

[30] Yamashita T, Forgues M, Wang W, Kim JW, Ye Q (2008). EpCAM and α-fetoprotein expression defines novel prognostic subtypes of hepatocellular carcinoma.. Cancer Res.

[31] Villanueva A, Hoshida Y, Toffanin S, Lachenmayer A, Alsinet C (2010). New strategies in hepatocellular carcinoma: genomic prognostic markers.. Clin Cancer Res.

[32] Mareel M, Leroy A (2003). Clinical, cellular, and molecular aspects of cancer invasion.. Physiol Rev.

[33] Bricard G, Cesson V, Devevre E, Bouzourene H, Barbey C (2009). Enrichment of human CD4+ V(alpha)24/Vbeta11 invariant NKT-cells in intrahepatic malignant tumors.. J Immunol.

