# Supplementary figures and images for: Immunological and Molecular Correlates of Disease Recurrence after Liver Resection for Hepatocellular Carcinoma
Source: PLoS One. 2012 Mar 2;7(3):e32493. doi: 10.1371/journal.pone.0032493 (PMC3292571; doi:10.1371/journal.pone.0032493)

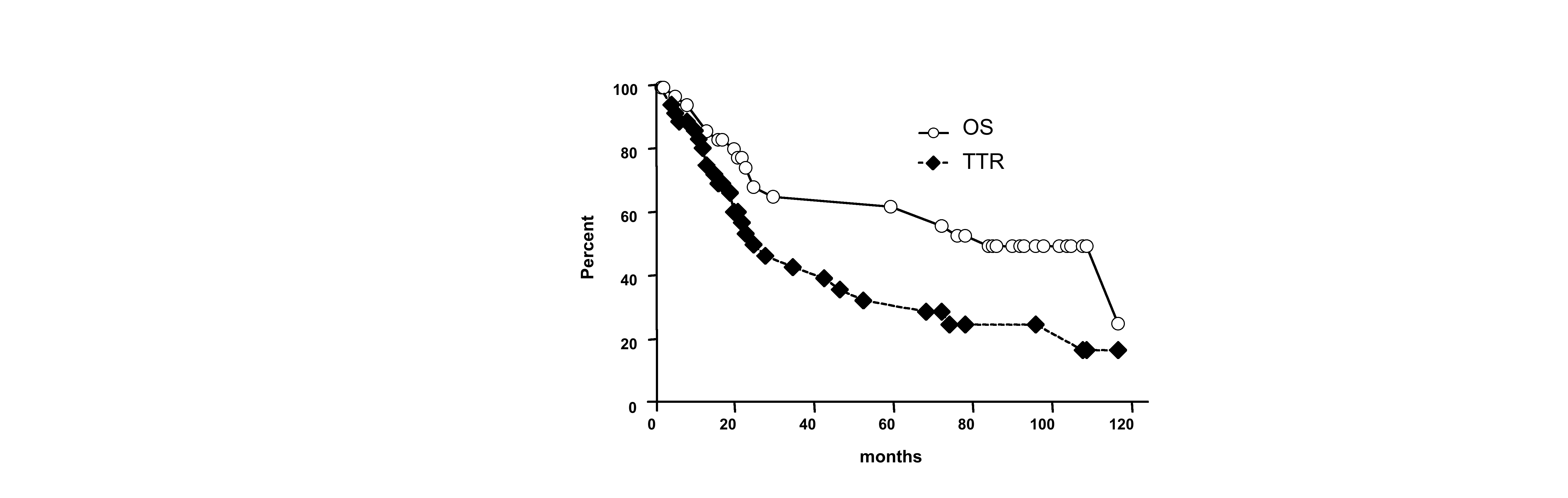

Supplement: Figure S1 — Percent survival of the whole study population after surgery. OS: overall survival; TTR: time to recurrence. (TIF) [file pone.0032493.s001.tif]
